# Supplementary material for: From pixels to phenotypes: Integrating image-based profiling with cell health data as BioMorph features improves interpretability
Source: Mol Biol Cell. 2024 Feb 2;35(3):mr2. doi: 10.1091/mbc.E23-08-0298 (PMC10916876; doi:10.1091/mbc.E23-08-0298)
Supplement: Supplementary file 1 [file mbc-35-mr2-s001.pdf]

Supplemental Materials

Molecular Biology of the Cell

Seal *et al*.

Supplementary Information

From Pixels to Phenotypes: Integrating Image-Based Profiling with Cell Health Data Improves Interpretability

Srijit Seal[1,2], Jordi Carreras-Puigvert[3], Shantanu Singh[1], Anne E Carpenter[1], Ola Spjuth[3], Andreas Bender*[2]*

[1]Imaging Platform, Broad Institute of MIT and Harvard, Cambridge MA, USA

[2]Yusuf Hamied Department of Chemistry, University of Cambridge, Cambridge, United Kingdom

[3]Department of Pharmaceutical Biosciences and Science for Life Laboratory, Uppsala University, Uppsala, Sweden

Cell Painting; Cell Health; BioMorph, Toxicity Prediction; Cell Morphology; Interpretability; Image-based profiling

* Email: ola.spjuth@farmbio.uu.se, ab454@cam.ac.uk

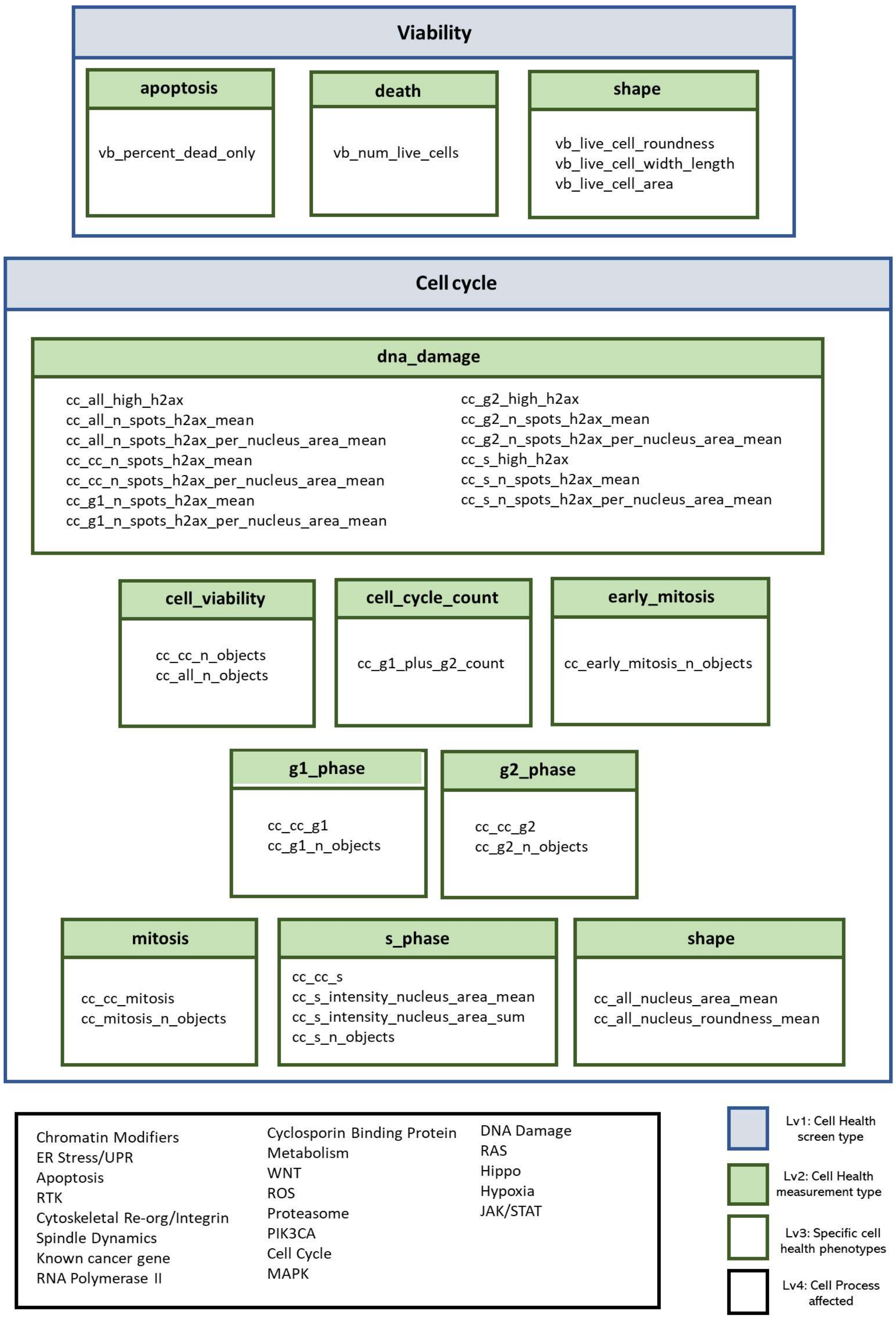

Figure S1: BioMorph terms' hierarchy with relationships for (a) Level 1: Cell Health assay type, (b) Level 2: Cell Health measurement type, (c) Level 3: Specific Cell Health phenotypes, (d) Level 4: Cell process affected (representative of morphological characteristics)

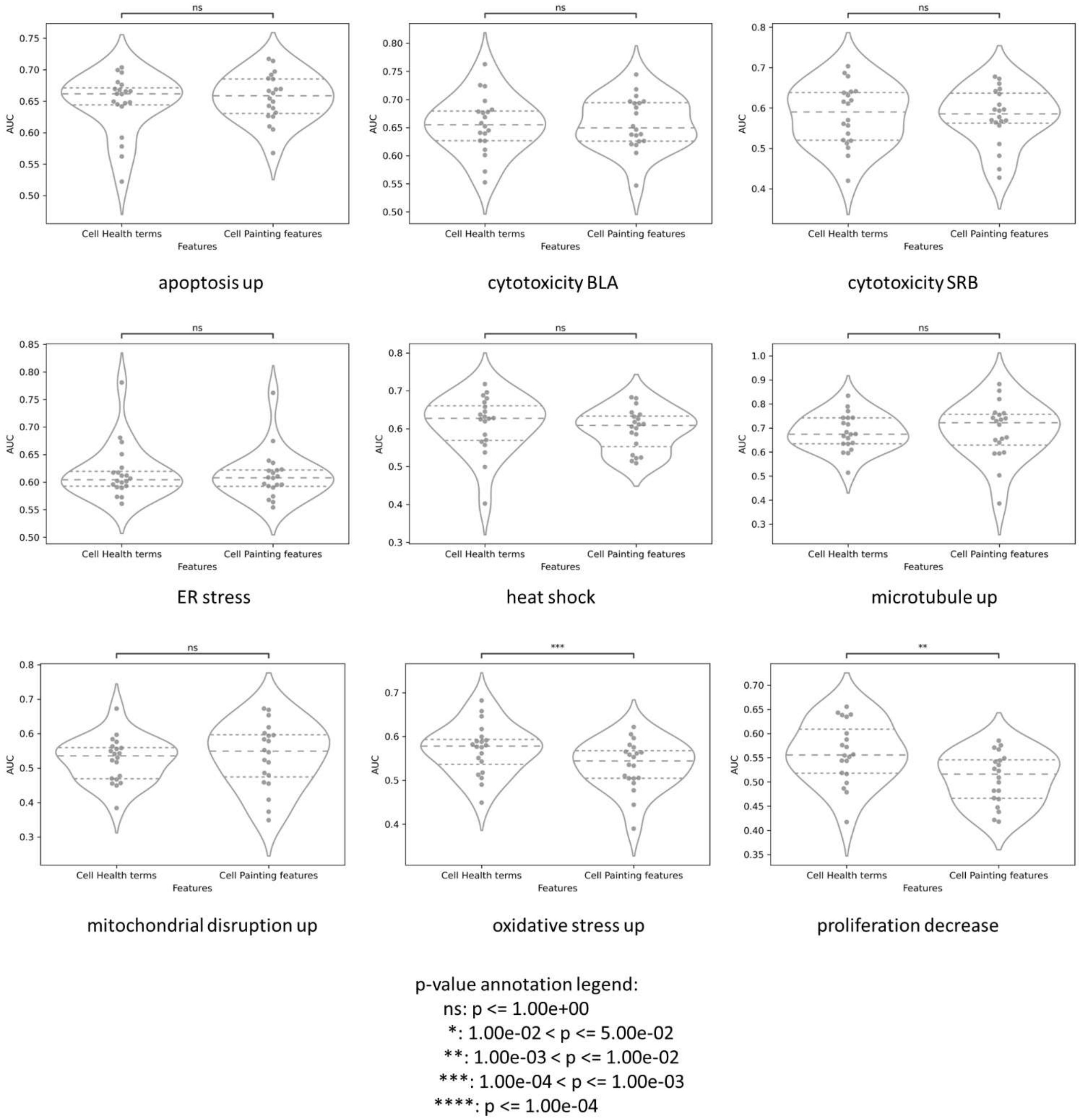

Figure S2: Distribution AUC for both models, namely, Cell Painting features and BioMorph terms as features when predicting 9 different broad biological activities in 5 times repeated 4-fold nested cross-validation.
