# Supplementary material for: From pixels to phenotypes: Integrating image-based profiling with cell health data as BioMorph features improves interpretability
Source: Mol Biol Cell. 2024 Feb 2;35(3):mr2. doi: 10.1091/mbc.E23-08-0298 (PMC10916876; doi:10.1091/mbc.E23-08-0298)
Supplement: Supplementary file 2 [file mbc-35-mr2-s002.pdf]

From Pixels to Phenotypes: Integrating Image-Based Profiling with Cell Health Data Improves Interpretability

Srijit Seal, Jordi Carreras-Puigvert, Shantanu Singh, Anne Carpenter, Ola Spjuth, and Andreas Bender

Corresponding author(s): Ola Spjuth, Uppsala University

Review Timeline:

Submission Date:	2023-08-01
Editorial Decision:	2023-09-04
Revision Received:	2023-09-11
Editorial Decision:	2023-10-03
Revision Received:	2023-12-06
Accepted:	2023-12-19

Editor-in-Chief: Matthew Welch

Transaction Report:

(Note: With the exception of the correction of typographical or spelling errors that could be a source of ambiguity, letters and reports are not edited. The original formatting of letters and referee reports may not be reflected in this compilation.)

RE: Manuscript #E23-08-0298
TITLE: From Pixels to Phenotypes: Integrating Image-Based Profiling with Cell Health Data Improves Interpretability

Dear Dr. Seal:

Reviewer 2 raises a significant issue that has to be addressed in a constructive manner. After this is done, I will send the manuscript to this reviewer for the second look. please also address other helpful comments of both reviewers.

Sincerely,

Alexander Mogilner
Monitoring Editor
Molecular Biology of the Cell

Dear Dr. Seal,

The review of your manuscript, referenced above, is now complete. The Monitoring Editor has decided that your manuscript is not acceptable for publication at this time, but may be deemed acceptable after specific revisions are made, as described in the Monitoring Editor's decision letter above and the reviewer comments below.

A reminder: Please do not contact the Monitoring Editor directly regarding your manuscript. If you have any questions regarding the review process or the decision, please contact the MBoC Editorial Office (mboc@ascb.org).

When submitting your revision include a rebuttal letter that details, point-by-point, how the Monitoring Editor's and reviewers' comments have been addressed. (The file type for this letter must be "rebuttal letter"; do not include your response to the Monitoring Editor and reviewers in a "cover letter.") Please bear in mind that your rebuttal letter will be published with your paper if it is accepted, unless you have opted out of publishing the review history.

Authors are allowed 90 days to submit a revision. If this time period is inadequate, please contact us at mboc@ascb.org.

Revised manuscripts are assigned to the original Monitoring Editor whenever possible. However, special circumstances may preclude this. Also, revised manuscripts are often sent out for re-review, usually to the original reviewers when possible. The Monitoring Editor may solicit additional reviews if it is deemed necessary to render a completely informed decision.

If your manuscript contains a Significance Statement, please make sure that it consists of three separate bullet points totaling a maximum of 100 words and addressing each of the following points (see https://www.molbiolcell.org/curation-tools):
First bullet: What is the background context? What gap in knowledge does this study addres?
Second bullet: What are the key findings? What is unique or new about the approach?
Third bullet: Why is this paper significant? How might it influence future research?

In preparing your revised manuscript, please follow the instruction in the Information for Authors (www.molbiolcell.org/info-for-authors). In particular, to prepare for the possible acceptance of your revised manuscript, submit final, publication-quality figures with your revision as described.

To submit the rebuttal letter, revised manuscript, and figures, use this link: Link Not Available

MBoC offers the option to publish your work with immediate open access. Open access can increase the discoverability and usability of your research. If you would like to publish your paper with immediate open access but did not select this option during initial submission, please contact the editorial office at mbc@ascb.org.

Thank you for submitting your manuscript to MBoC. We look forward to receiving your revised paper.

Sincerely,

Eric Baker
Journal Production Manager
MBoC Editorial Office
mbc@ascb.org

Reviewer #1 (Remarks to the Author):

This manuscript presents a proof-of-concept study aiming to integrate a comprehensive set of image-based metrics with an output of cell health assays. The authors sought to link subsets of morphological measures with multi-leveled biological characteristics to generate a set of biologically interpretable descriptors called BioMorph terms. They demonstrated how mapping imaging data onto BioMorph space can help uncover relationships between molecular perturbations, affected cellular processes, and the development of specific phenotypes, ultimately enabling the generation of mechanistic hypotheses. Although this paper doesn't offer a novel biological insight, it presents convincing examples of how the BioMorph pipeline can be used to gain such insights. All parts of the manuscript are carefully written, with a clear, logical presentation of the background, rationale, results, limitations, and an extensive description of the method. Given that this is a proof-of-concept paper, I do not see any major issues with its current form. Below, please find my minor suggestions for improving readability:

1. Page 8: "to predict which these sets of selected" should be "to predict which of the sets of selected"

2. Page 8: abbreviation MCC is used for the first time but not defined.

3. Page 9: "a morphological change that includes information about the "fraction of caspase negative in dead cells" (level 3) associated with apoptosis (level 1), measurement type (level 2), and the effect of CRISPR knockout". I believe it should be "a morphological change that includes information about the "fraction of caspase negative in dead cells" (level 3) associated with apoptosis (level 2), cell viability (level 1), and the effect of CRISPR knockout"

4. Page 10: AUC-ROC is used here for the first time but defined much later at the end of the Methods section.

5. Page 10: "repeated nester cross-validation". A reference would be helpful here.

6. Page 13: "Similarly, the most enriched processes for the other endpoints (Figure 5), i.e. Cytotoxicity BLA (Hippo signalling pathway), Cytotoxicity SRB (cyclosporine binding protein), heat shock response (DNA damage), oxidative stress (apoptosis and hypoxia), and proliferation (Hippo pathways), are all in agreement". For consistency with the terminology, I would change this as "Similarly, the most enriched processes for the other endpoints (Figure 5), i.e., Hippo signaling pathway for Cytotoxicity BLA, cyclosporine binding protein for Cytotoxicity SRB, DNA damage for heat shock response, apoptosis and hypoxia for oxidative stress, and Hippo pathways for proliferation, are all in agreement"

7. Page 18: "Error: Bookmark not defined". Apparently, there is a problem with the reference here.

8. Page 19: "a wide range of feature measurements (share, area, size, correlation, texture etc)." Should be shape, not share.

9. Page 19: the abbreviation InChI should be defined.

10. Page 22: "using the J statistic value". A reference would be helpful here.

11. Page 23: "First, we next used". Should be "First, we used" or "We next used"

Reviewer #2 (Remarks to the Author):

Summary: The authors present a computational approach to map cell morphology features to another space that represents phenotypes or other interpretable features.

General comments: While the paper certainly presents a study by skilled researchers, it appears quite outdated with its complete reliance on expert designed features. The approach is novel, but should be better embedded into related approaches. Technically, there appear to be no major flaws.

Major comments:
A) The relevance is very limited because of its reliance on outdated computer

vision methods.
While there are hundreds of thousands of scientific works now relying on image features from deep learning systems, this work still builds on expert-designed features.
This felt immediately anachronistic and a simple literature search immediately brought forward a large number of papers suggesting microscopy image encoders that provide rich features (see at least references [1-5]). For those features that are much richer than expert-designed ones, equipping them with interpretability would even be more beneficial and make the approach much more relevant.
The authors should re-do a similar analysis with features from a pre-trained deep learning system, compare the results to the computer vision features, and elaborate on the improved interpretablity of deep learning systems for microscopy.

B) Novelty: This appears to be novel and original work that has not been carried out before. However, the authors are not embedding their work well into other related works and are very focused on presenting their own approach. Has a similar effort (connecting features from some modality with some more interpretable other entities), been done before? How? What are the closest related works in this area? How are they different?
The authors should elaborate on that in detail in the introduction section.

C) The computational steps performed for this work are well described in the Supplementary. However, the processing steps are difficult to follow in the main manuscript. Figure 2 is trying to explain the steps, but is not overly informative. The authors should improve Figure 2 and connect it better with the descriptions in the text.
A concrete suggestion is to add boxes to each of the steps (A, B, C, D) that provide more information.

Minor comments and typos:
- The term "BioMorph" is dropped upon the reader and unclear at first. I understood that this is what the authors try to introduce/define. I would suggest to make this clearer already in the abstract, e.g. "We propose a new space, which we call BioMorph, which [...]", or something along those lines.
- p 18 top: missing bookmark.
- p 5 bottom: "Heath" --> "Health"

References:

[1] Stuckner, J., Harder, B., & Smith, T. M. (2022). Microstructure segmentation with deep learning encoders pre-trained on a large microscopy dataset. NPJ Computational Materials, 8(1), 200.
[2] Khadangi, A., Boudier, T., & Rajagopal, V. (2021, January). EM-net: Deep learning for electron microscopy image segmentation. In 2020 25th international conference on pattern recognition (ICPR) (pp. 31-38). IEEE.
[3] Cross-Zamirski, J. O., Mouchet, E., Williams, G., Schönlieb, C. B., Turkki, R., & Wang, Y. (2022). Label-free prediction of cell painting from brightfield images. Scientific reports, 12(1), 10001.
[4] Sanchez-Fernandez, A., Rumetshofer, E., Hochreiter, S., & Klambauer, G. (2022). CLOOME: contrastive learning unlocks bioimaging databases for queries with chemical structures. bioRxiv, 2022-11.
[5] Wong, D. R., Logan, D. J., Hariharan, S., Stanton, R., Clevert, D. A., & Kiruluta, A. (2023). Deep representation learning determines drug mechanism of action from cell painting images. Digital Discovery.

Dear Editor,

Many thanks for the very positive comments. Please find attached our reply to the points raised by the reviewers:

Editor and Reviewer Comments:

Reviewer #1 (Remarks to the Author):

This manuscript presents a proof-of-concept study aiming to integrate a comprehensive set of image-based metrics with an output of cell health assays. The authors sought to link subsets of morphological measures with multi-leveled biological characteristics to generate a set of biologically interpretable descriptors called BioMorph terms. They demonstrated how mapping imaging data onto BioMorph space can help uncover relationships between molecular perturbations, affected cellular processes, and the development of specific phenotypes, ultimately enabling the generation of mechanistic hypotheses. Although this paper doesn't offer a novel biological insight, it presents convincing examples of how the BioMorph pipeline can be used to gain such insights. All parts of the manuscript are carefully written, with a clear, logical presentation of the background, rationale, results, limitations, and an extensive description of the method.

Given that this is a proof-of-concept paper, I do not see any major issues with its current form. Below, please find my minor suggestions for improving readability:

1. Page 8: "to predict which these sets of selected" should be "to predict which of the sets of selected"

Thank you for this correction. We have modified the sentence as suggested.

2. Page 8: abbreviation MCC is used for the first time but not defined.

We apologize for this oversight. We have now defined MCC as "Matthews Correlation Coefficient" the first time it is mentioned in the text.

3. Page 9: "a morphological change that includes information about the "fraction of caspase negative in dead cells" (level 3) associated with apoptosis (level 1), measurement type (level 2), and the effect of CRISPR knockout". I believe it should be "a morphological change that includes information about the "fraction of caspase negative in dead cells" (level 3) associated with apoptosis (level 2), cell viability (level 1), and the effect of CRISPR knockout"

We appreciate your attention to detail and agree with your proposed correction. We have revised this section to reflect the levels correctly as suggested.

4. Page 10: AUC-ROC is used here for the first time but defined much later at the end of the Methods section.

We have addressed this by replacing AUC-ROC with “Area Under Curve-Receiver Operating Characteristic (AUC)” when it is first introduced on Page 10.

5. Page 10: "repeated nester cross-validation". A reference would be helpful here.

Thank you for pointing this out. We have added a reference to elucidate the concept of repeated nested cross-validation ([31] Parvandeh S, Yeh HW, Paulus MP, McKinney BA (2020) Consensus features nested cross-validation. Bioinformatics 36:3093–3098.)

6. Page 13: "Similarly, the most enriched processes for the other endpoints (Figure 5), i.e. Cytotoxicity BLA (Hippo signalling pathway), Cytotoxicity SRB (cyclosporine binding protein), heat shock response (DNA damage), oxidative stress (apoptosis and hypoxia), and proliferation (Hippo pathways), are all in agreement". For consistency with the terminology, I would change this as "Similarly, the most enriched processes for the other endpoints (Figure 5), i.e., Hippo signaling pathway for Cytotoxicity BLA, cyclosporine binding protein for Cytotoxicity SRB, DNA damage for heat shock response, apoptosis and hypoxia for oxidative stress, and Hippo pathways for proliferation, are all in agreement"

We appreciate your suggestion for improving clarity and consistency in our terminology. The sentence has been revised as recommended.

7. Page 18: "Error: Bookmark not defined". Apparently, there is a problem with the reference here.

Our apologies for the oversight. We have rectified this error and ensured that the reference ([6] Way GP, Kost-Alimova M, Shibue T, et al. Predicting cell health phenotypes using image-based morphology profiling. Mol Biol Cell. 2021;32(9):995-1005) is correctly cited.

8. Page 19: "a wide range of feature measurements (share, area, size, correlation, texture etc)." Should be shape, not share.

Thank you for catching that typo. We have made the necessary correction.

9. Page 19: the abbreviation InChI should be defined.

We've now defined InChI as "International Chemical Identifier" upon its first mention.

10. Page 22: "using the J statistic value". A reference would be helpful here.

We have supplemented this statement with the original reference ([75] Youden WJ (1950) Index for rating diagnostic tests. Cancer 3:32–35) to provide context for the J statistic value.

11. Page 23: "First, we next used". Should be "First, we used" or "We next used"

We have amended the sentence to "First, we used" for better clarity and coherence.

Reviewer #2 (Remarks to the Author):

Summary: The authors present a computational approach to map cell morphology features to another space that represents phenotypes or other interpretable features.

General comments: While the paper certainly presents a study by skilled researchers, it appears quite outdated with its complete reliance on expert designed features. The approach is novel, but should be better embedded into related approaches. Technically, there appear to be no major flaws.

Thank you for recognizing the novelty of our work. While we acknowledge the prominence of deep learning techniques in image processing, our approach has its foundations in classic image-processing features for specific reasons as described below.

Major comments:

A) **The relevance is very limited because of its reliance on outdated computer vision methods. While there are hundreds of thousands of scientific works now relying on image features from deep learning systems, this work still builds on expert-designed features. This felt immediately anachronistic and a simple literature search immediately brought forward a large number of papers suggesting microscopy image encoders that provide rich features (see at least references [1-5]). For those features that are much richer than expert-designed ones, equipping them with interpretability would even be more beneficial and make the approach much more relevant. The authors should re-do a similar analysis with features from a pre-trained deep learning system, compare the results to the computer vision features, and elaborate on the improved interpretablity of deep learning systems for microscopy.**

We appreciate the reviewer raising this concern, as other readers may also wonder the same: why not use deep learning-based features rather than classical image-processing features? This is an excellent question, and we did not explain this in the paper. The omission of an explanation for our choice of classical image-processing features over deep learning features was an oversight in our initial submission. Some of our co-authors are at the forefront of developing and testing deep learning-based feature extractors in image-based profiling experiments. [59, 60, 62, 63, 64, 66] In light of the reviewer's feedback, we have incorporated a comprehensive section in the Limitations elaborating on our choice (pg. 16):

"Despite the overall success of deep learning for segmentation and feature extraction [62-65], such approaches have generally shown performance in the same range as classical image features in the domain of image-based profiling. A few exceptions have emerged very recently, when specialized training strategies have allowed deep learning features to improve somewhat, up to a ~20% improvement in various tasks [63,66]. This improvement was found to be inconsistent across datasets [66] and hence both types of features need to be compared using ground truth on each new dataset,

which often is unavailable. Further, for many applications, the loss of interpretability for deep-learned features outweighs the performance improvement. Finally, given how rapidly moving is the research in this area, a 'standard' method for extracting features using deep learning does not yet exist, such that a new BioMorph space would need to be mapped/defined for each set of deep learned features. For these reasons, we focused our study on classical image features, building the foundation for future studies on deep learning-based features, once a canonical set of such features is defined by the community. Our results lend confidence that this approach would likely be fruitful and would, to some degree, overcome the interpretability limitation of deep learning-based features."

We hope this addition adequately addresses the concerns surrounding our methodological choices.

(59) Pawlowski N, Caicedo JC, Singh S, et al (2016) Automating Morphological Profiling with Generic Deep Convolutional Networks. bioRxiv 4–8. https://doi.org/10.1101/085118

(60) Caicedo JC, Arevalo J, Piccioni F, et al (2022) Cell Painting predicts impact of lung cancer variants. Mol Biol Cell 33:ar49. https://doi.org/10.1091/mbc.E21-11-0538

(62) Lafarge MW, Caicedo JC, Carpenter AE, et al (2019) Capturing Single-Cell Phenotypic Variation via Unsupervised Representation Learning. Proc Mach Learn Res 102:315–325

(63) Moshkov N, Bornholdt M, Benoit S, et al (2022) Learning representations for image-based profiling of perturbations. bioRxiv 2022.08.12.503783. https://doi.org/10.1101/2022.08.12.503783

(64) Chow YL, Singh S, Carpenter AE, Way GP (2022) Predicting drug polypharmacology from cell morphology readouts using variational autoencoder latent space arithmetic. PLoS Comput Biol 18:e1009888. https://doi.org/10.1371/journal.pcbi.1009888

(65) Wong DR, Logan DJ, Hariharan S, et al (2023) Deep representation learning determines drug mechanism of action from cell painting images. Digit Discov. https://doi.org/10.1039/d3dd00060e

(66)Kim V, Adaloglou N, Osterland M, et al (2023) Self-supervision advances morphological profiling by unlocking powerful image representations. bioRxiv 2023.04.28.538691. https://doi.org/10.1101/2023.04.28.538691

B) Novelty: This appears to be novel and original work that has not been carried out before. However, the authors are not embedding their work well into other related works and are very focused on presenting their own approach. Has a similar effort (connecting features from some modality with some more interpretable other entities), been done before? How? What are the closest related works in this area? How are they different? The authors should elaborate on that in detail in the introduction section.

We appreciate the reviewer suggesting this. We have expanded the introduction to better contextualize our work among related studies (pg. 5).

"Recent advancements in data integration methodologies have demonstrated the potential of connecting distinct data modalities to enhance interpretability. This is common in gene set enrichment analysis where methods such as the $\chi 2$ test have been used which combine a set of gene expression features connected by annotations to a common pathway into a gene-set level statistic.[22] Another example is the Gene Ontology transformed gene expression profiles of small molecule perturbations developed using Principal Angle Enrichment Analysis (PAEA).[23],[24] Other studies have combined prior knowledge of pathways and gene expression data to identify latent variables (inferred using models) to elucidate underlying patterns in gene sets that are unique compared to the input gene expression data.[25] The application of contrastive learning has also emerged, such as CLOOME,

aiming to bridge the gap between image-based representations and chemical structures by embedding them into the same representation space.[26] In this context, our work introduces a method specifically tailored for Cell Painting, with an emphasis on data-based feature grouping. Unlike extant approaches that predominantly aim to improve target prediction, our methodology aims to establish a novel interpretative space, facilitating a deeper comprehension of cellular biology phenomena."

(22) Hung JH, Yang TH, Hu Z, et al (2012) Gene set enrichment analysis: Performance evaluation and usage guidelines. Brief Bioinform 13:281–291. https://doi.org/10.1093/bib/bbr049

(23) Clark NR, Szymkiewicz M, Wang Z, et al (2015) Principal Angle Enrichment Analysis (PAEA): Dimensionally reduced multivariate gene set enrichment analysis tool. Proc - 2015 IEEE Int Conf Bioinforma Biomed BIBM 2015 2015:256–262. https://doi.org/10.1109/BIBM.2015.7359689

(24) Wang Z, Clark NR, Ma'ayan A (2016) Drug-induced adverse events prediction with the LINCS L1000 data. Bioinformatics 32:2338–2345. https://doi.org/10.1093/bioinformatics/btw168

(25) Basili D, Reynolds J, Houghton J, et al (2022) Latent Variables Capture Pathway-Level Points of Departure in High-Throughput Toxicogenomic Data. Chem Res Toxicol 35:670–683. https://doi.org/10.1021/acs.chemrestox.1c00444

(26) Klambauer G, Sanchez Fernandez A, Rumetshofer E, Hochreiter S (2022) CLOOME: contrastive learning unlocks bioimaging databases for queries with chemical structures. bioRxiv 0–17. https://doi.org/10.1101/2022.11.17.516915

C) The computational steps performed for this work are well described in the Supplementary. However, the processing steps are difficult to follow in the main manuscript. Figure 2 is trying to explain the steps, but is not overly informative. The authors should improve Figure 2 and connect it better with the descriptions in the text. A concrete suggestion is to add boxes to each of the steps (A, B, C, D) that provide more information.

We've revised Figure 2, enhancing its descriptive nature and ensuring better alignment with the manuscript's text.

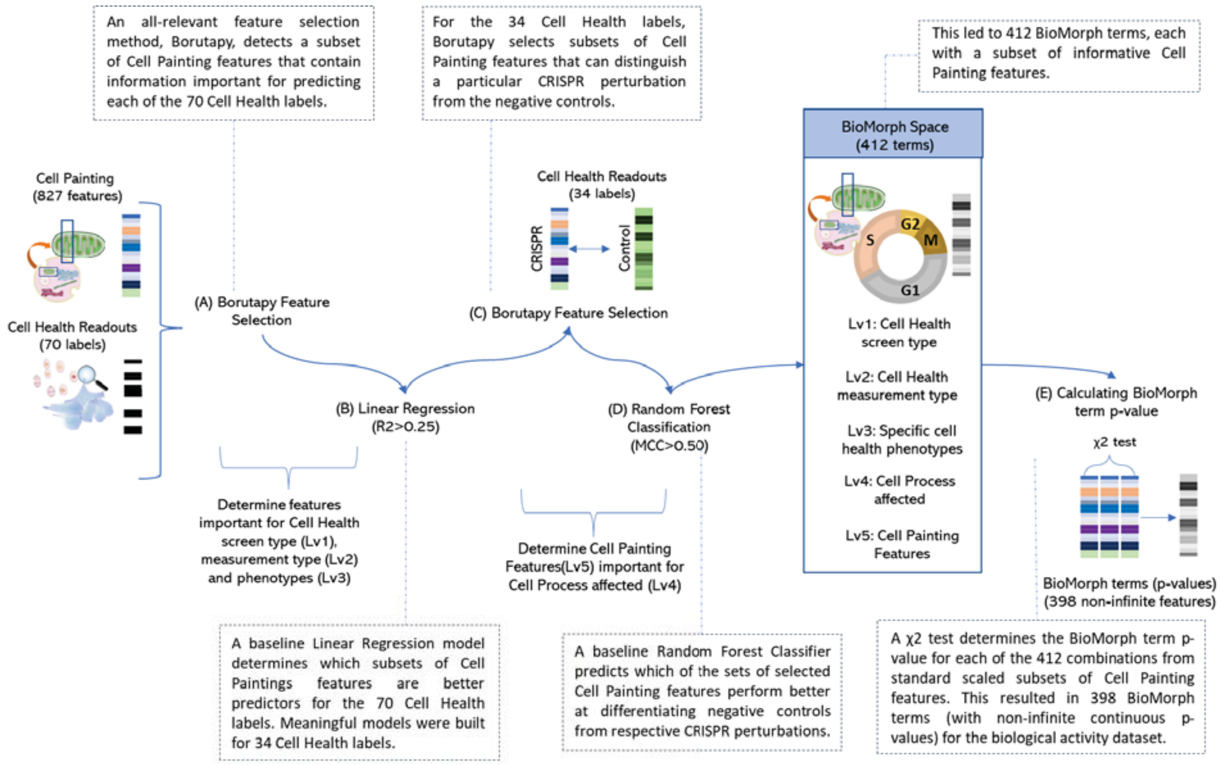

Figure 2. Schematic representation of methodology to generate BioMorph terms mapped from CRISPR perturbations measured by the Cell Painting assay and Cell Health assay. Further details on all terms are in Supplementary Figure S1 with all BioMorph Space listed in Supplementary Table S1.

Minor comments and typos:

- The term "BioMorph" is dropped upon the reader and unclear at first. I understood that this is what the authors try to introduce/define. I would suggest to make this clearer already in the abstract, e.g. "We propose a new space, which we call BioMorph, which [...]", or something along those lines.

We have amended the abstract and introduced "BioMorph" more clearly,

Abstract: “In this study, we propose a new feature space, which we call BioMorph, that maps specific Cell Painting features with readouts from comprehensive Cell Health assays.”

Introduction (pg. 6): We propose a new feature space, called the BioMorph space, that provides a function-informed framework for interpreting Cell Painting features in the cell biology context.

- p 18 top: missing bookmark.

This has been rectified. The correct reference is now included.

- p 5 bottom: "Heath" --> "Health"

Thank you for catching this. The error has been corrected.

References:

[1] Stuckner, J., Harder, B., & Smith, T. M. (2022). Microstructure segmentation with deep learning encoders pre-trained on a large microscopy dataset. NPJ Computational Materials, 8(1), 200.

[2] Khadangi, A., Boudier, T., & Rajagopal, V. (2021, January). EM-net: Deep learning for electron microscopy image segmentation. In 2020 25th international conference on pattern recognition (ICPR) (pp. 31-38). IEEE.

[3] Cross-Zamirski, J. O., Mouchet, E., Williams, G., Schönlieb, C. B., Turkki, R., & Wang, Y. (2022). Label-free prediction of cell painting from brightfield images. Scientific reports, 12(1), 10001.

[4] Sanchez-Fernandez, A., Rumetshofer, E., Hochreiter, S., & Klambauer, G. (2022). CLOOME: contrastive learning unlocks bioimaging databases for queries with chemical structures. bioRxiv, 2022-11.

[5] Wong, D. R., Logan, D. J., Hariharan, S., Stanton, R., Clevert, D. A., & Kiruluta, A. (2023). Deep representation learning determines drug mechanism of action from cell painting images. Digital Discovery.

We hope the revisions address the concerns and further elucidate the reasoning behind our choices. We look forward to your continued consideration.

2nd Editorial Decision October 3, 2023

RE: Manuscript #E23-08-0298R
TITLE: From Pixels to Phenotypes: Integrating Image-Based Profiling with Cell Health Data Improves Interpretability

Dear Dr. Spjuth:

The reviewer, who is an expert in the field, found the revisions insufficient. Normally, we allow just one round of revision, but after I reviewed the reviewer's comments, I think that if you do exactly what the reviewer advises, the study could become acceptable. If you are ready to do that, please resubmit, and I will ask the reviewer to take another look.

Sincerely,

Alexander Mogilner
Monitoring Editor
Molecular Biology of the Cell

Dear Dr. Spjuth,

The review of your manuscript, referenced above, is now complete. The Monitoring Editor has decided that your manuscript is not acceptable for publication at this time, but may be deemed acceptable after specific revisions are made, as described in the Monitoring Editor's decision letter above and the reviewer comments below.

A reminder: Please do not contact the Monitoring Editor directly regarding your manuscript. If you have any questions regarding the review process or the decision, please contact the MBoC Editorial Office (mboc@ascb.org).

When submitting your revision include a rebuttal letter that details, point-by-point, how the Monitoring Editor's and reviewers' comments have been addressed. (The file type for this letter must be "rebuttal letter"; do not include your response to the Monitoring Editor and reviewers in a "cover letter.") Please bear in mind that your rebuttal letter will be published with your paper if it is accepted, unless you have opted out of publishing the review history.

Authors are allowed 90 days to submit a revision. If this time period is inadequate, please contact us at mboc@ascb.org.

Revised manuscripts are assigned to the original Monitoring Editor whenever possible. However, special circumstances may preclude this. Also, revised manuscripts are often sent out for re-review, usually to the original reviewers when possible. The Monitoring Editor may solicit additional reviews if it is deemed necessary to render a completely informed decision.

In preparing your revised manuscript, please follow the instruction in the Information for Authors (www.molbiolcell.org/info-for-authors). In particular, to prepare for the possible acceptance of your revised manuscript, submit final, publication-quality figures with your revision as described.

If your manuscript contains a Significance Statement, please make sure that it consists of three separate bullet points totaling a maximum of 100 words and addressing each of the following points (see https://www.molbiolcell.org/curation-tools):
First bullet: What is the background context? What gap in knowledge does this study addres?
Second bullet: What are the key findings? What is unique or new about the approach?
Third bullet: Why is this paper significant? How might it influence future research?

To submit the rebuttal letter, revised manuscript, and figures, use this link: Link Not Available

MBoC offers the option to publish your work with immediate open access. Open access can increase the discoverability and usability of your research. If you would like to publish your paper with immediate open access but did not select this option during initial submission, please contact the editorial office at mbc@ascb.org.

Thank you for submitting your manuscript to MBoC. We look forward to receiving your revised paper.

Sincerely,

Eric Baker
Journal Production Manager
MBoC Editorial Office
mbc@ascb.org

Reviewer #2 (Remarks to the Author):

Summary: The rebuttal has addressed a few of my concerns, but my main concern about the limited relevance because of the use of outdated computer vision methods remains. The problem has even aggravated because an extremely biased view on the research landscape and its results is brought forward with many tenuous or false claims. The statements of the authors contradict hundreds of publications in computer vision presenting overwhelming evidence that learned features from deep networks outperform handcrafted features.

Major concerns:

1) Tenuous and incorrect claims; biased view on the research results;
In the new or rewritten paragraph on p.17 ("Despite ..") and extremely biased view with many tenuous or incorrect statements is brought forward. Scientific writing should be objective and embed works into the full landscape of related works.
a) "such approaches have generally shown performance in the same range as classical image features in the domain of image-based profiling". This statement is incorrect. A clear perspective on the performance of methods is revealed when investigating scientific challenges [1,2] or benchmark datasets [3] because of their unbiased evaluation. All recent scientific challenges on microscopy images of cells have been won by Deep Learning methods, with not a single CellProfiler-based method ranking anywhere near the Top10 (see, e.g., Refs [1] and [2, Table 1]). Benchmarks are also exclusively dominated by Deep Learning methods. Also in many other studies Deep Learning methods by far outperform CellProfiler (see, e.g., [5,6]).
The authors should revise the statement and clearly state that there is overwhelming evidence for the strong performance of Deep Learning methods over handcraftet features for computer vision tasks, such as microscopy image classification.
b) "the loss of interpretability of deep-learning features outweights the performance improvement". This is incorrect or tenuous, because the deep learning features can be better interpreted (using e.g. GradCAM), than most of the CellProfiler features. Furthermore, it is incorrect because even a tiny performance improvement can substantially increase screening outcome, which can save time- and monetary costs, which greatly outweighs the alleged interpretability loss.
Furthermore, this also contradicts the whole purpose of the study, because the study aims at making features interpretable.
The authors should revise their statement and support it by citing relevant publications. The authors should provide a clear quantative metric that determines how performance improvement can be traded-off against interpretability. The authors should present an appropriate metric to quantify the interpretability of a method, if they want to claim that the CellProfiler features are more interpretable than deep networks.

If an appropriate unbiased perspective on related works on microscopy imaging tasks is presented, I would still find this paper suitable for publication and recommend its acceptance even if the study is not extended to Deep Learning features.

References.
[1] Ouyang, W., Winsnes, C. F., Hjelmare, M., Cesnik, A. J., Åkesson, L., Xu, H., ... & Lundberg, E. (2019). Analysis of the human protein atlas image classification competition. Nature methods, 16(12), 1254-1261.

[2] Le, T., Winsnes, C. F., Axelsson, U., Xu, H., Mohanakrishnan Kaimal, J., Mahdessian, D., ... & Lundberg, E. (2022). Analysis of the human protein atlas weakly supervised single-cell classification competition. Nature Methods, 19(10), 1221-1229.
[3] Hörst, F., Rempe, M., Heine, L., Seibold, C., Keyl, J., Baldini, G., ... & Kleesiek, J. (2023). CellViT: Vision Transformers for Precise Cell Segmentation and Classification. arXiv preprint arXiv:2306.15350.
[4] Zhang, D., Song, Y., Liu, D., Jia, H., Liu, S., Xia, Y., ... & Cai, W. (2018). Panoptic segmentation with an end-to-end cell R-CNN for pathology image analysis. In Medical Image Computing and Computer Assisted Intervention-MICCAI 2018: 21st International Conference, Granada, Spain, September 16-20, 2018, Proceedings, Part II 11 (pp. 237-244). Springer International Publishing.
[5] Pärnamaa, T., & Parts, L. (2017). Accurate classification of protein subcellular localization from high-throughput microscopy images using deep learning. G3: Genes, Genomes, Genetics, 7(5), 1385-1392.
[6] Wong, D. R., Logan, D. J., Hariharan, S., Stanton, R., Clevert, D. A., & Kiruluta, A. (2023). Deep representation learning determines drug mechanism of action from cell painting images. Digital Discovery.

RE: Manuscript #E23-08-0298RR
TITLE: "From Pixels to Phenotypes: Integrating Image-Based Profiling with Cell Health Data Improves Interpretability"

Dear Dr. Spjuth:

I am pleased to accept your manuscript for publication in Molecular Biology of the Cell.

Sincerely,
Alexander Mogilner
Monitoring Editor
Molecular Biology of the Cell

Dear Dr. Spjuth:

Congratulations on the acceptance of your manuscript! Thank you for choosing to publish your work in Molecular Biology of the Cell (MBoC).

Within 10 days, an unedited PDF of your manuscript will be published on MBoC in Press, an early release version of the journal. The date your manuscript appears on this site is the official publication date.

Your copyedited and typeset manuscript will be scheduled for publication in the next available issue of MBoC. Our production team will notify you when the page proofs of your paper are ready for your review.

MBoC offers the option to publish your paper with immediate open access. Open access can increase the discoverability and usability of your research. If you would like to publish your paper with immediate open access but did not select this option during initial submission, please contact the MBoC Editorial Office (mbc@ascb.org).

We invite you to submit images related to your accepted manuscript to be considered for the journal cover. Please contact mboc@ascb.org to learn how to submit candidate cover images.

Authors of Articles and Brief Communications are also encouraged to create a short video abstract to accompany their article when it is published. These video abstracts, known as Science Sketches, are up to 2 minutes long and will be published on YouTube and then embedded in the article abstract. Science Sketch Editors on the MBoC Editorial Board will provide guidance as you prepare your video. Information about how to prepare and submit a video abstract is available at www.molbiolcell.org/science-sketches. Please contact mboc@ascb.org if you are interested in creating a Science Sketch.

We look forward to publishing your paper in MBoC.
Sign up for MBoC's new content alerts here https://tinyurl.com/3bcsyk3e

Sincerely,

Eric Baker
Journal Production Manager
MBoC Editorial Office
mbc@ascb.org

Reviewer #2 (Remarks to the Author):

My comments and concerns have been addressed. I now recommend acceptance of this manuscript.
